# Successful treatment of discoid lupus erythematosus with tapinarof 1% cream monotherapy

**DOI:** 10.1016/j.jdcr.2024.09.010

**Published:** 2024-09-30

**Authors:** Kareena S. Garg, Leonardo Tjahjono

**Affiliations:** a Department of Dermatology, Georgetown University School of Medicine, Washington, District of Columbia; b Department of Dermatology, George Washington School of Medicine, Washington, District of Columbia

**Keywords:** aryl hydrocarbon receptor, cutaneous lupus erythematous, discoid lupus erythematous, tapinarof

## Introduction

Discoid lupus erythematosus (DLE) is a common type of chronic cutaneous lupus erythematosus.^1^ It is an autoimmune condition that presents as erythematous, scaly plaque(s) that are typically photodistributed; it can progress to permanent scarring and hyper and hypopigmentation.^1^ Treatment options include ultrapotent topical steroids, topical calcineurin inhibitors, topical Janus-Kinase inhibitors, antimalarial agents, and other immunosuppressants.^1^ We present a case of a patient with recalcitrant DLE who was successfully treated with tapinarof 1% cream monotherapy.

## Case report

**Fig 1:**
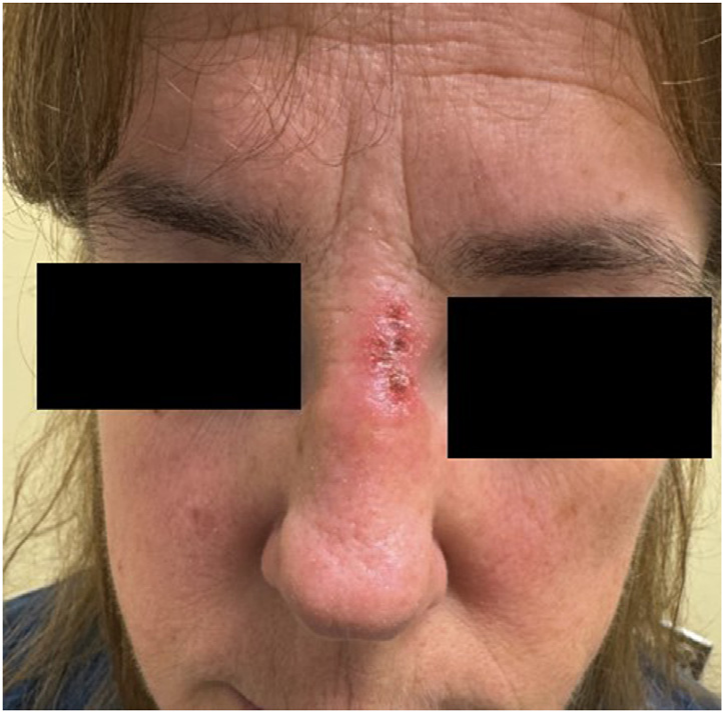
Initial DLE presentation with indurated and ulcerated erythematous plaque on the nasal bridge. *DLE*, Discoid lupus erythematosus.

**Fig 2:**
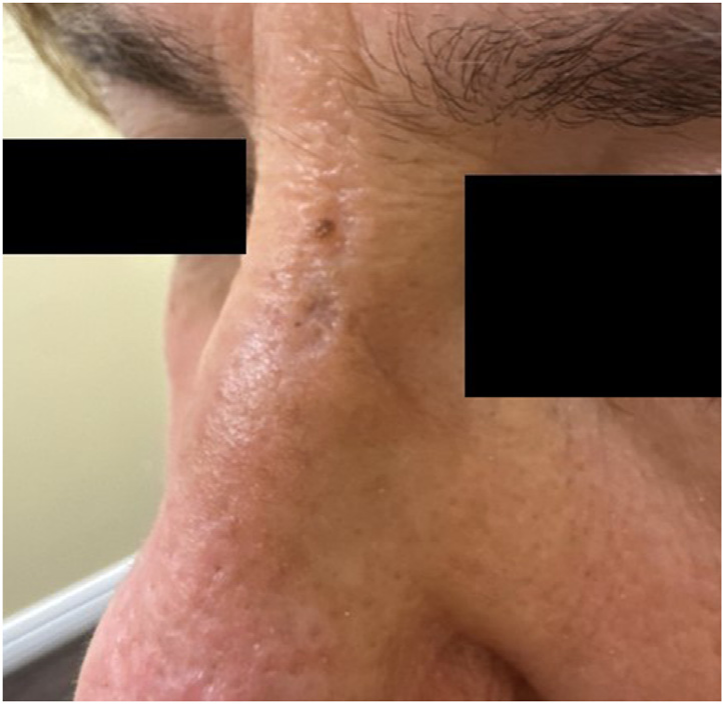
Resolution of DLE following application of once-a-day tapinarof 1% cream for 3 weeks. *DLE*, Discoid lupus erythematosus.

A 56-year-old woman with no significant past medical history presented to the clinic with indurated and ulcerating erythematous plaques and severe burning sensation on her nasal bridge and frontal scalp for 3 months duration. Biopsy showed interface dermatitis and superficial and deep periadnexal lymphocytic infiltrates, supportive for the diagnosis of DLE. Laboratory assessments were not supportive for systemic lupus erythematosus (SLE). Treatment was attempted with tacrolimus 0.1% ointment and clobetasol 0.05% ointment twice a day for 4 weeks each with continued worsening. The patient declined any systemic treatment and intralesional steroids injection; ruxolitinib 1.5% cream was unobtainable. Off-label use of tapinarof 1% cream samples once a day for 3 weeks provided rapid improvement of the burning sensation with complete resolution of the plaque (Figs 1 and 2). There were no adverse reactions while using the tapinarof 1% cream. Remission was sustained at 3-month follow-up without further medication use.

## Discussion

Tapinarof is Food and Drug Administartion–approved for psoriasis and well-known to provide disease remission for up to 4 months once the psoriatic lesions are fully cleared.^2^ To our knowledge, this was the first report of rapid successful treatment and sustained disease remission of DLE with tapinarof 1% cream.

DLE often relies on early interventions, both topical and systemic agents, to prevent permanent scarring. Tapinarof is an aryl hydrocarbon receptor agonist that leads to downregulation of proinflammatory cytokines such as interleukin 17 and interleukin 22.^3^^,^^4^ Additionally, aryl hydrocarbon receptor regulates the differentiation and balance of CD4+ T helper 1, 2, 17, follicular, and regulatory immune cells, which are central in development of autoimmune diseases like SLE and DLE.^5^^,^^6^

In a recent study, systemic intraperitoneal administration of tapinarof in mice with SLE strains showed successful amelioration of SLE phenotypes.^5^ The study demonstrated that aryl hydrocarbon receptor-agonist agent tapinarof inhibited the phosphorylation of the JAK2-STAT3 pathway, which subsequently suppressed T follicular helper cell differentiation. It also reduced the imbalanced proportion of T helper 1/2 cells. Tapinarof also increased T regulatory cell development.^5^ In all, tapinarof regulatory effects on aforementioned cells developments and cytokines lead to immunosuppressive effects and decrease autoantibodies responsible for SLE.^5^ This may explain mechanism of action of successful use of tapinarof cream in our DLE patient.

While promising, additional controlled studies are needed to evaluate tapinarof as a safe and effective treatment option for DLE.

## Conflicts of interest

Dr Leonardo Tjahjono has served as a consultant for Bristol Myers Squibb.
